# Insight into phytase-producing microorganisms for phytate solubilization and soil sustainability

**DOI:** 10.3389/fmicb.2023.1127249

**Published:** 2023-04-11

**Authors:** Sheikh Rizwanuddin, Vijay Kumar, Pallavi Singh, Bindu Naik, Sadhna Mishra, Mansi Chauhan, Per Erik Joakim Saris, Ankit Verma, Vivek Kumar

**Affiliations:** ^1^Department Food Science and Technology, Graphic Era (Deemed to be University), Dehradun, India; ^2^Himalayan School of Biosciences, Swami Rama Himalayan University, Dehradun, India; ^3^Department of Biotechnology, Graphic Era (Deemed to be University), Dehradun, India; ^4^Faculty of Agricultural Sciences, GLA University, Mathura, India; ^5^Department of Microbiology, Graphic Era (Deemed to be University), Dehradun, India; ^6^Department of Microbiology, Faculty of Agriculture and Forestry, University of Helsinki, Helsinki, Finland

**Keywords:** phosphorus, microbial phytase, bioinoculants, transgenic, growth inducer, soil sustainability, agriculture, nutrient cycle

## Abstract

The increasing demand for food has increased dependence on chemical fertilizers that promote rapid growth and yield as well as produce toxicity and negatively affect nutritional value. Therefore, researchers are focusing on alternatives that are safe for consumption, non-toxic, cost-effective production process, and high yielding, and that require readily available substrates for mass production. The potential industrial applications of microbial enzymes have grown significantly and are still rising in the 21st century to fulfill the needs of a population that is expanding quickly and to deal with the depletion of natural resources. Due to the high demand for such enzymes, phytases have undergone extensive research to lower the amount of phytate in human food and animal feed. They constitute efficient enzymatic groups that can solubilize phytate and thus provide plants with an enriched environment. Phytases can be extracted from a variety of sources such as plants, animals, and microorganisms. Compared to plant and animal-based phytases, microbial phytases have been identified as competent, stable, and promising bioinoculants. Many reports suggest that microbial phytase can undergo mass production procedures with the use of readily available substrates. Phytases neither involve the use of any toxic chemicals during the extraction nor release any such chemicals; thus, they qualify as bioinoculants and support soil sustainability. In addition, phytase genes are now inserted into new plants/crops to enhance transgenic plants reducing the need for supplemental inorganic phosphates and phosphate accumulation in the environment. The current review covers the significance of phytase in the agriculture system, emphasizing its source, action mechanism, and vast applications.

## Introduction

**Figure 1:**
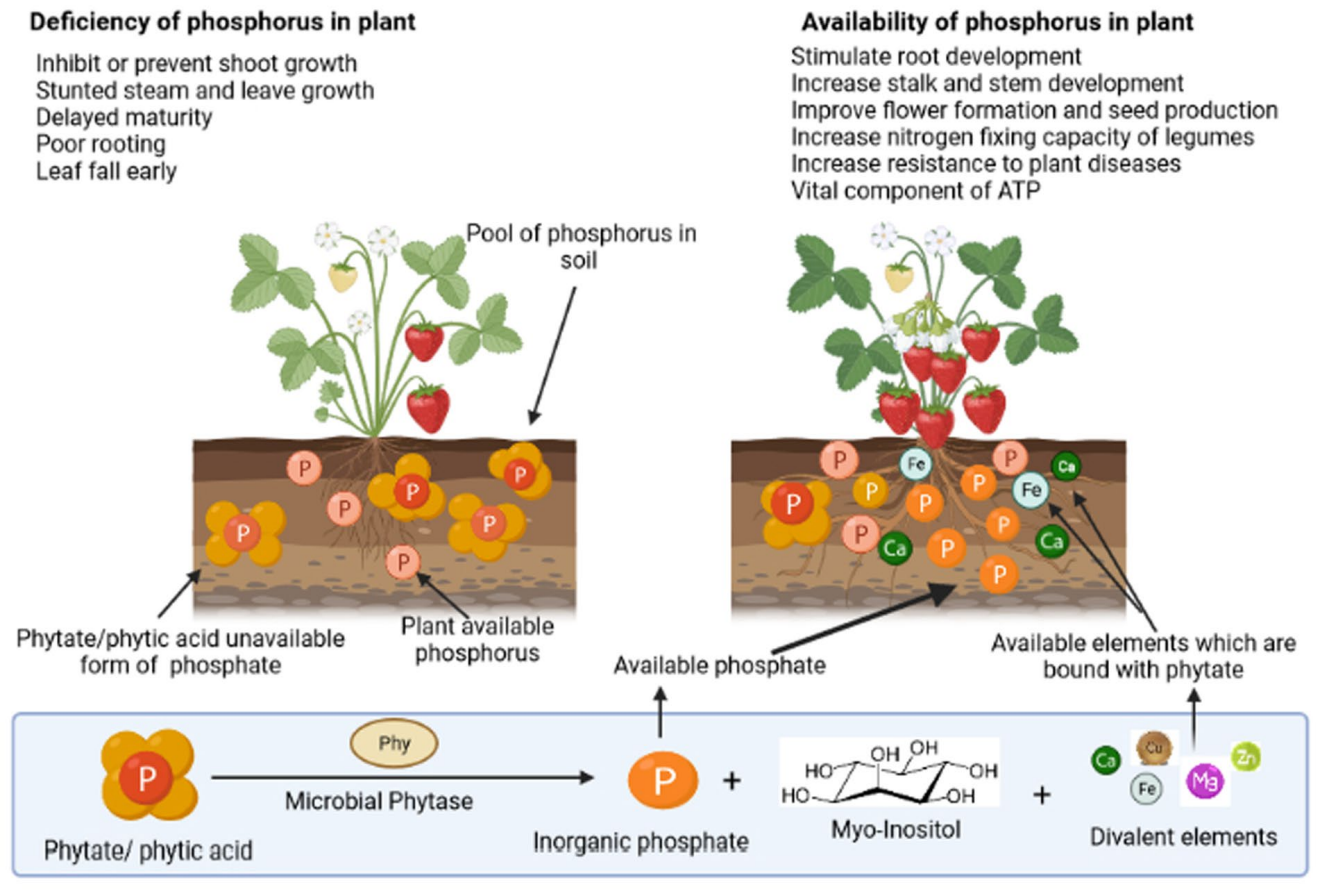
Depiction of the importance of phosphorus to a plant and the role of phosphate solubilizing microorganisms in the utilization of different forms of phosphorus *via* diverse processes.

Phosphorus (P) is one of the Earth’s (lithosphere) less-abundant macronutrients (0.1% of total), and soil phosphorus concentration depends on the phosphorus content of the parent material. Organic phosphorus (P_o_) constitutes approximately 30%–65% of the total phosphorus; however, inorganic phosphorus (P_i_) accounts for 30%–75% of the total soil phosphorus (Fujita et al., 2017; Wu et al., 2022). Its availability throughout the initial stages of plant development is vital for the establishment of plant reproductive component primordia. It is essential for boosting root strength and ramification, giving plant vigor and pathogen resistance. Moreover, it aids in the production of seeds and the early maturity of crops such as grains and legumes (Sharma et al., 2013). Figure 1 demonstrates the various routes of phosphorus utilization by the plants in the soil. Plants require phosphorus for their various fundamental processes such as photosynthesis, flowering, fruiting, and maturation. Significantly high phosphorus levels are required for cell division and in the development of meristematic tissues. It also promotes the growth of roots and helps in nitrogen fixation (Weil and Brady, 2017). While in the case of phosphorus deficiency, the plant is typically spindly, thin-stemmed, and stunted and has dark and almost bluish-green foliage, instead of light foliage. Therefore, phosphorus-deficient plants frequently appear relatively normal unless much larger and healthy plants are present to provide a comparison. In addition, delayed maturation, irregular flowering, and poor seed quality are traits of phosphorus-deficient plants. A significant phosphorus deficit can result in senescence and withering of leaves. Lack of phosphorus causes many plants to exhibit purple colors in their leaves and stems. Phosphorus is found aggregated in the form of myo-inositol hexabisphosphate in soil, which is chemically known as phytate/phytic acid. Soil phytate can be produced through microbial soil P_i_ transformation, plant tissues, and monogastric animal manures (Liu et al., 2022). Phytate mineralization is observed by many microorganisms and can be implemented in plant systems for induced agricultural sustainability. The previous study demonstrated that the exogenous addition of phytate-rich substrates and soil immobilization and transformation by P fertilizers are associated with phytate accumulation (Menezes-Blackburn et al., 2013).

Phytate is a chemical derivative of inositol (myo-inositol hexabisphosphate) and is the most widely distributed form of phosphorus in the soil (Duong et al., 2018). It is present mainly in the bound form with other minerals and significantly contributes to the organic soil phosphate pool (P_o_) (Gerke, 2015). Structurally, it is a six-hydroxyl alcohol with phosphoric acid (six molecules) residues bound to its hydroxyl groups.

As the metallic cations of Ca, Fe, K, Mg, Mn, and Zn are tightly bound by the negatively charged phosphate in PA, they become insoluble and are hence not available for plants (Azeem et al., 2014; Sun et al., 2021). Small quantities of inorganic phosphorus added to the rhizosphere might act as a stimulator to phytic acid mineralization, thus improving plant phosphorus feeding (Elhaissoufi et al., 2022). However, the phytate present in the soil cannot be directly utilized by the plants. For soil phytate to contribute to plant P nutrition, phosphate ester (C-O-P), phosphoanhydride (P-O-P), or phosphonate (C-P) must first be dephosphorylated through phytase-mediated hydrolysis.

Phytases are abundantly found in nature. The primary sources include plants, microbes (bacteria and fungi), and some animal tissues (Cookson, 2002; Konietzny and Greiner, 2002). Due to their catalytic properties and simplicity of enzyme production, phytases of microbial origin are the most suitable for use in the commercial biotechnological production of enzymes. A few plant roots have been detected with phytases having low hydrolytic activity and not secreting phytase into the rhizosphere. Therefore, plants are unable to utilize soil phytates by their mechanism. Phytases are actively excreted by microscopic soil fungi and, to a lesser extent, by bacteria. Plant roots were shown to have weak phytase activity, but since the enzyme is not released into the rhizosphere, plants are unable to absorb the fixed phosphorus from soil phytates on their own (Kashirskaya et al., 2020).

Phytases are enzymes that catalyze the hydrolysis of phytic acid in different positions of the inositol ring and release phosphorus, zinc, and other minerals in inorganic form, thereby increasing the absorption of minerals by plants (Vats and Banerjee, 2004). The physicochemical properties of phytases affect their stability, mobility, and ability to hydrolyze soil phytate. Moreover, their enormous anion-holding capacity and surface area suggest that inositol phosphates have a strong affinity for soil colloids. Their activity is dependent on the soil type. Azeem et al. (2014) reported that clays and organic materials restrict enzyme activity more than diverse soil surfaces, with great specificity to soil characteristics and mineralogy. Moreover, after 28 days of phytase adsorption in sandy soil, 40% of the extra phytase remained active, but only 5% remained active in soil with higher clay percentages. A well-aerated soil will allow for faster phosphorus solubilization than saturated, wet soil. Interlaminar gaps in clay minerals reduced phytase activities more than 1:1 phyllosilicate.

Because of their global commercial importance, sources of phytases in microbes are rated more essential than other sources (Singh et al., 2020). Although plants cannot obtain phosphorus from phytate directly, the existence of phosphate-solubilizing microorganisms in the rhizosphere can compensate for this loss (Kaur, 2020). Microbial phytases were more effective than those extracted from plants (Azeem et al., 2014). Currently, microbial phytase is a highly researched area known to counter food toxicity and security concerns. A report in support of this fact has been published currently by Ladics et al. (2020). They studied the toxicity effects of PhyG (bacterial phytase variant) developed *via* fermentation using *Trichoderma reesei* and observed non-toxic when consumed on remarkably higher doses of consumption in broilers. Another study by Thorsen et al. (2021) evaluated the safety of Phytase HM derived from *Aspergillus oryzae*. They studied the effects *in vivo* and observed no mutagenic or inflammatory effects. Significantly, these produced positive responses to the growth and bone health of poultry animals.

## Phytate: Organic phosphorus

Phosphorus deposition as phytate within soils is approximately 50 million metric tonnes annually, accounting for 65% of phosphorus fertilizer. Inositol hexakisphosphate (IP6), a chemical derived from phytate, was found in 1903 and is the primary P storage form in many soil and plant tissues (Azeem et al., 2014). Soil phytate may derive from plant tissues, monogastric animal manures, and microbial conversion from soil Pi. Plants and bacteria both produce phytate, although plants are the primary producer. Phytate exists in six inositol esters, i.e., Mono-, bis-, tris-, tetrakis-, pentakis-, and hexakis-phosphates (IP1–6), out of which, IP6 is the major form, making up to 83%–100% of IP (Hill et al., 2007). Moreover, IP6 exits in four stereoisomers with their abundance in the order: myo (56–90%) > scyllo (20%–50%) > Dchiro (6%–10%,) > neo (1%–5%) (Turner et al., 2012). IP6 stereoisomer is mostly contributed by plants while the rest of the stereoisomers are synthesized by microbes present in the soil. Phytate has a high degree of charge density and hence has strong interaction with soil and is responsible for binding Fe/Al-oxides in acidic soil and Ca/Mg minerals in alkaline soil. Due to pH dependency, and numerous hydroxyl- and oxo- groups on the surface, phytate develops a chelating affinity with mineral cations and forms phytate-mineral complexes. However, multiple hydroxyl groups allow phytic acid and its deprotonated phytate forms to form strong inter- and intra-molecular hydrogen bonds and aid in solubility and acidity in aqueous solutions (Liu et al., 2022). Despite the possibility of phytate being present in soil solution, there is not any proven record that plants directly absorb phytate from the soil. The soil phytate must first be dephosphorylated from phosphate ester (COP), phosphoanhydride (POP), or phosphonate (CP) *via* phytase-mediated hydrolysis to contribute to plant P nutrition.

## Mechanisms of phytate solubilization

Phytate has a high affinity for soil; therefore, it gets accumulated in the soil as compared to other esters of phosphorus. Hence, its availability is low, thereby interfering with the interaction with phytase which reduces the cleavage of esters bonds of phytate and mineralization of the inositol ring (Tang et al., 2006). There are two approaches to improving phytate access by phytase: desorption and solubilization. Protons, organic acids, and phenolic acids can all desorbate or solubilize P in soil, with organic acids being the main solubilizer of the rarely available phosphorus (Richardson et al., 2011). There are three methods through which the carboxylate groups in organic acids can mobilize phytate. First, by substituting P with a carboxylate anion, carboxylates can desorb phosphate anions from the soil by ligand exchange. Due to its higher number of carboxyl groups and closer pK2 value (4.76 vs. 4.28) to soil pH (4.5–9.5), tribasic citrate releases more P than dibasic oxalate and causes oxalate to degrade more quickly (Menezes-Blackburn et al., 2016). Second, carboxylates can remove P sorption sites by solubilizing Fe and Al *via* H+. Finally, they can dissolve organic matter (OM) that binds to P *via* Fe/Al-bridges, releasing phosphate as the OM-Fe/Al-P complex (Gerke, 2010). Phytate solubilization in the soil is improved by chelating metals bound in metal-phytate complexes to release P and chelating metals to form complexes that bind to soils and prevent microbial degradation of organic acids (Gerke, 2015). Microbes can efficiently decompose organic acids in the soil solution, but their decomposition is slowed significantly by sorption onto the soil. For phytate to dissolve in soil, organic acids must be present in the soil solution and Gerke provided a summary of the impact of organic acids on plants’ uptake of phytate-P (Gerke et al., 2000; Gerke, 2015).

**Figure 2:**
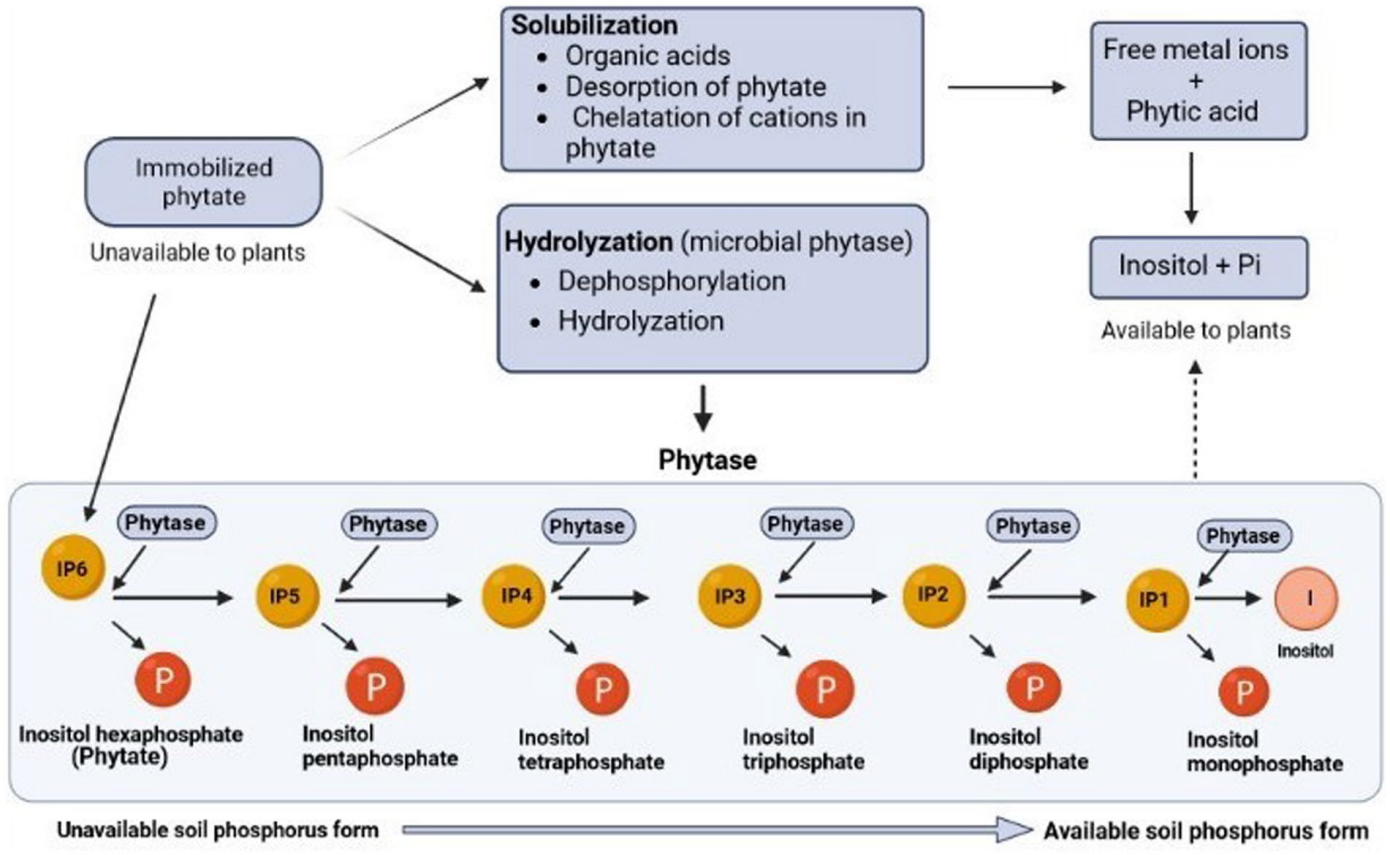
The various processes of phytate solubilization highlighting the breakdown of phytate (unavailable form) into simpler compounds (available form).

Both the plant and microbial phytases play a significant role in the solubilization of phytate (Figure 2) present in the soil. Phytases catalyze the mineralization, or the conversion of organic phosphorus from phytate to inorganic phosphorus, which can be easily absorbed by plants (Ariza et al., 2013). The extracellular phytase secreted from the roots is crucial for soil phytate hydrolysis when there is a lack of P, these either stay attached to the cell walls of the roots or are discharged straight into the rhizosphere to catalyze phytate hydrolysis (Sun et al., 2022). By using genetic engineering tools, transgenic plants have been developed to express phytase genes of microbial origin to break down soil phytate. This improves plant phosphorus accumulation and increases biomass (Xiao et al., 2005; Balaban et al., 2017).

## Factors regulating phytate solubilization

The plant can only absorb the inorganic phosphate produced by the hydrolysis of phytic acid. In various physicochemical circumstances, the phosphate ester bond in phytate is relatively stable. The negative charge of phytate is responsible for interaction with metals present in soil and the formed metal complexes affect its solubility (Celi et al., 2001). Metal phytate complexes are soluble as follows: in terms of metal species, Na, Ca, and Mg are preferred over Cu, Zn, Mn, Cd, and Al over Fe. In terms of pH, pH 5.0 is preferred over pH 7.5. Except for Al-phytate, most phytate-metal complexes resist acid hydrolysis, and dry complexes are typically stable at high pressure and temperature (except Ca phytate) (De Boland et al., 1975). At all pH ranges, phytate combines with calcium to form soluble complexes (Ca_1_- or Ca_2−_ phytate) or insoluble precipitates such as Ca_3−_ phytate. Strong chelating chemicals like EDTA are insufficient to dissolve complexes of metals without first reducing the metals (like Fe) (Sun et al., 2021). Phytate may undergo severe immobilization, which prevents it from being hydrolyzed by phytase, resulting in its limited availability and significant accumulation in soils. This is supported by the fact that phytate has a higher reactivity than P_i_ and other P_o_ compounds (Celi and Barberis, 2005). The organic acids produced by plant exudates and microbes also affect the solubility of phytate in soil (Richardson et al., 2011). The phytate solubility in the soil is also dependent on the phytase enzyme. The activity of phytase is affected by soil pH and is optimum in the range of 2.5–8.0 and then decreases with an increase in pH. In addition, because of its sorption onto soil minerals like montmorillonite, phytase action is suppressed (Leprince and Quiquampoix, 1996). *Aspergillus niger* phytase varied in its ability to prevent metal complexes from being hydrolyzed by enzymes; however, it has been discovered that the Fe phytate complex showed the most outstanding inhibition. Strong chelating chemicals like EDTA are insufficient to dissolve complexes of metals without first reducing the metals (like Fe) (Sun et al., 2021). Moreover, the interactions among phytate-mineralizing bacteria, bacteria-eating nematodes, and mycorrhizal fungi also boost plant P uptake from phytate in soils with significant P adsorption (Ranoarisoa et al., 2020).

## Types of phytases and their sources

**Figure 3:**
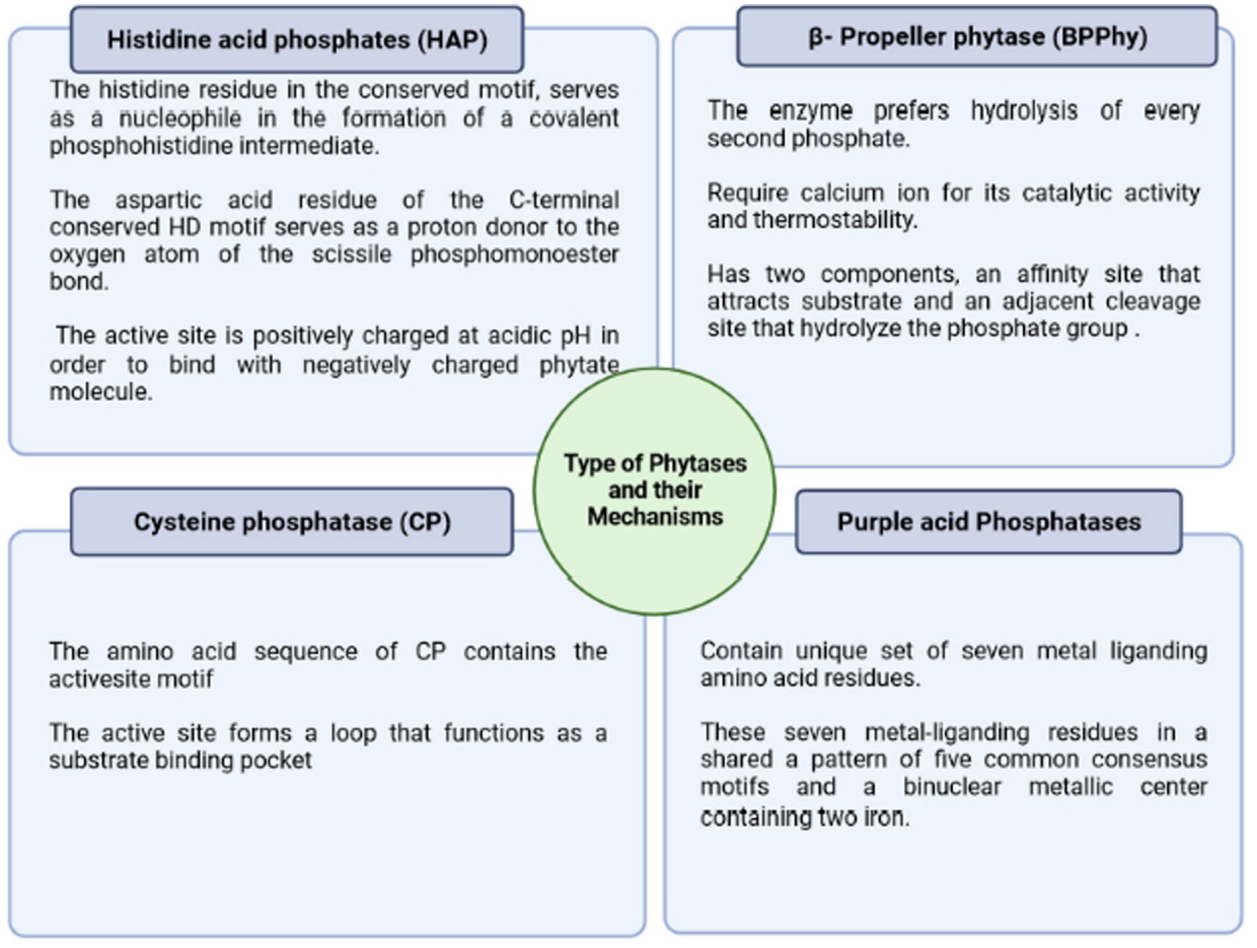
Major types of phytases and their different mechanism of action stating a basic criterion for their classification.

Phytases were discovered in 1907 and are considered among the 10 most significant discoveries in agricultural processes during the last century. Phytase enzymes are phosphatases that may initiate the progressive dephosphorylation of phytate (Figure 3). They are classified according to their source, pH optimal (alkaline or acid phytases), and current catalytic processes. Although not all phytases have the exact catalytic mechanism, there are four separate categories of these enzymes with diverse structures and processes (Lei et al., 2013; Menezes-Blackburn et al., 2013) *viz.*, β-propeller phytase (BPPs), protein tyrosine phosphatase-like phytase (PTP-like phytase), purple acid phosphatase (PAPs), and last histidine acid phosphatases (HAPhy). Whereas HAPhy is further subdivided into multiple inositol polyphosphate phosphatases (MINPs) due to the observed difference in sequence homology. Cysteine phosphatase-like phytase (CP-phytase) and BPP are exclusively found in microbes, but HAP and PAPhy phytases have been found in plants. Because of the economic relevance of these enzymes, the structure and characteristics of microbial HAPs have been intensively researched (Madsen and Brinch-Pedersen, 2020). Furthermore, the catalytic process is related to molecular structure, which fluctuates substantially across and within different categories. On the other hand, the structural differences among phytases *via* the phytate dephosphorylation on the inositol ring at varying locations (3, 5, and 6) categorize it into 3-phytase, 5-phytase, and 6-phytase (Singh B. et al., 2018; Rix et al., 2022).

Because certain phytases are intracellular but may not participate in external phosphorus-phytate dephosphorylation, soil and manure phytase activity cannot be linked entirely to P nutrition. Rhizosphere phytase activity may enhance plant development in soils with inadequate P supplies (Liu et al., 2022). Although, this phytase activity is commonly regarded as a direct indication of the metabolic needs of the microbial population in several situations and circumstances. Xenobiotic phosphonates (flame retardants, detergent additives, pesticides, and antibiotics) are today’s major effluents (Behera et al., 2014). Most organic molecules have high molecular weights and are often resistant to enzymatic hydrolysis; in such cases, phosphatases are one of the best-researched enzymes capable of biodegradation. Depending on the application, a phytase with commercial potential must meet several quality requirements. When added to feed, enzymes should be efficient in releasing phytate phosphate, stable to withstand heat inactivation from feed processing and storage, and inexpensive to produce. Since feed pelleting is frequently done at temperatures between 65°C and 95°C, thermostability is a crucial concern (Konietzny and Greiner, 2004).

## Source of phytase

**Figure 4:**
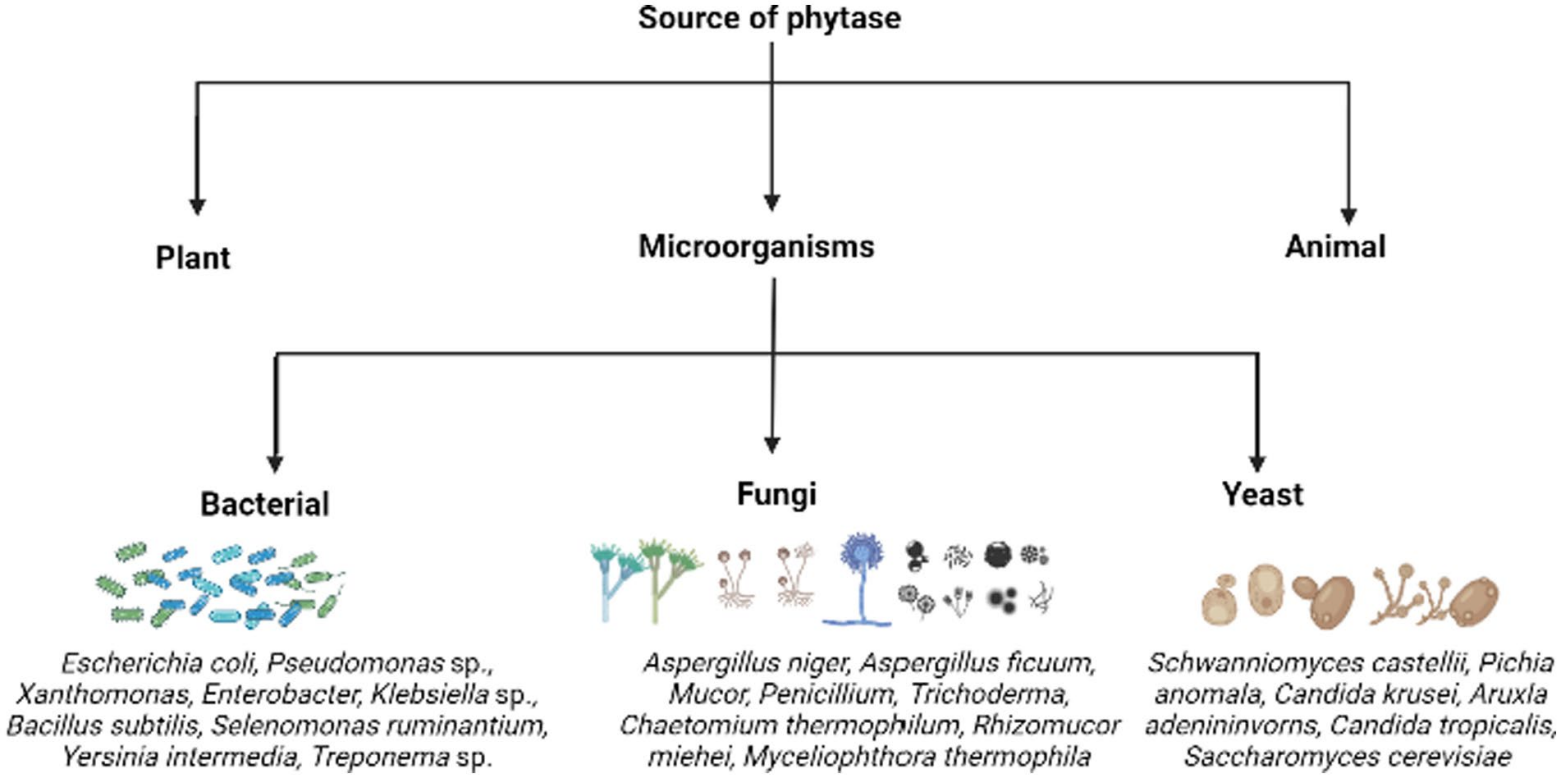
The various sources of phytases highlighting certain examples of phytate/phosphate solubilizing microorganisms.

Phytases are extensively distributed among various life forms. Plants, microorganisms, and animals are the sources used to obtain phytase (Figure 4). Among them, microbial phytase is the major source of phytases, produced by yeasts, bacteria, and fungi, followed by plants.

In plants, phytase may be found in higher concentrations in wheat, barley, and peas, while lower content in soybeans, maize, spinach, and so on. Phytase in the plant was first discovered in rice bran, which formed several phosphatidyl inositols as an intermediate or final product (Secco et al., 2017; Kumar and Sinha, 2018; Singh N. et al., 2018). Plant phytases can be MINPPs, PAPhys, or, in rare situations, HAPs, which are more similar to fungus HAPs than MINPPs. Plant phytases are typically produced during seed germination and can be expressed during grain filling. Phytases are usually linked with roots and are often extruded from them. Phytases exhibit wider mechanisms of action as discussed in the earlier sections, indicating their potential to work on other substrates. In plants, there is no obvious functional distinction between the phytase groups (Madsen and Brinch-Pedersen, 2020).

The majority of HAP family plant phytases begin phytate hydrolysis at position C6 of the myo-inositol hexaphosphate ring and are thus classified as type 6 phytases. Some plant phytases have been identified as alkaline or purple acid phosphatases. Plant phytase is inactive in dry seeds, but its activity increases during germination because phytase releases phosphorus to meet the plant’s needs (Hussain et al., 2022); also reported to inhibit the translocation of heavy metals in plants, for instance, *Pteris vittata* PvPHY1, a new root-specific phytase expressed in tobacco was reported to promote growth and P accumulation by 10%–50% (Sun et al., 2022). Plant-derived phytase enzymes are unstable at pH levels below 4.0 and above 7.0, but phytase enzymes derived from bacteria are stable at pH levels above 8.0 and below 3.0. The optimum temperature range for plant phytase is 40–60°C, while higher temperatures of 70–90°C result in plant phytase deactivation. When the temperature surpasses 80°C, pellet formation results in the inactivation of plant phytase (Hussain et al., 2022). The main issue with generating plant phytases is that a cost-effective and efficient method of synthesizing the enzymes has yet to be established. Plant phytases have lower pH and heat stability than microbial phytases. However, manufacturing phytase from plants is time-consuming, complicated, and costly. This is also not economically advantageous. Due to the tough plant cell walls and phyto-depositions, the extraction of phytases generated by plants necessitates the use of chemicals and takes more time than that of microbes. Moreover, the price of chemicals may change depending on the type of plant source. On the other hand, by using an optimized substrate, growth conditions, and manufacturing techniques, microorganisms may be employed for mass production. As a result, the production of phytase from microbial origin has more significant potential (Dailin et al., 2019; Alemzadeh et al., 2022).

## Microbial phytase

Microbial phytase activity was first discovered in *A. niger* mycelium more than a century ago (Gessler et al., 2018). Microbial phytases, preferably those derived from filamentous fungi such as *Mucor piriformis*, *Penicillium*, *Rhizopus*, *Aspergillus*, *Thermomyces*, and *Trichoderma*, constitute a large number of scientific reports published in response to this topic (Jatuwong et al., 2020). Fungi have more efficiency in phytate degradation due to certain factors, such as their hyphae can traverse longer distances in soil and extract P more efficiently and their ease of culture and excellent production yields (Shahryari et al., 2018). Fungi are also known to exhale a high concentration of organic acids, which act as a chelator and are considered the major process of inorganic phosphate solubilization. Microbial phytases do not require or release any toxic chemicals, thus they are safe biofertilizers and can also benefit farmers who practice organic farming (Gaind and Nain, 2015). Singh and Satyanarayana (2015) reported *Schwanniomyces castellii* as the most phytase-yielding strain. Several reports revealed the diversity of phytase-producing yeasts such as *Candida otropicalis, Candida krusei*, *Arxula adeninivorans*, *Debaryomyces castelii*, *Kluyveromyces fragilis*, *Kluyveromyces lactis*, *Schwanniomyces castellii*, *Zygosaccharomyces bisporus*, *Zygosaccharomyces priorionus*, and *P. spartinae* (Kaur et al., 2007; Menezes et al., 2020; Soman et al., 2020; Molina et al., 2021). Phytases have been reported in bacteria such as *Aerobacter aerogenes*, *Bacillus* sp. (*Bacillus licheniformis*, *Bacillus subtilis* P6, and *B. subtilis*) (Zhao et al., 2021; Trivedi et al., 2022), *Enterobacterium*, anaerobic rumen bacteria (*Megasphaera elsdenii* and *Selenomonas ruminantium*), *Escherichia coli*, *Lactobacillus amylovorus, and Pseudomonas* sp. *Lactobacillus sanfranciscensis*, which were discovered as the highest phytase producers among the lactic acid bacterial strains recovered from sour doughs (Konietzny and Greiner, 2004). Bacterial phytases have high thermostability. Other characteristics of bacterial phytases include comparatively smaller structures (Hussain et al., 2022), high substrate specificity, proteolysis resistance, and catalytic efficiency.

## Contribution of phytases in agriculture

Organic phosphorus, in the form of inositol phosphates, contributes 30%–80% of total phosphorus in soils. Phosphorus is inaccessible to plants due to interactions with reactive metals, such as Zn^2+^, Al^3+^, Cu^2+^, Ca^2+^, and Fe^2+^, and calcareous and normal soils (Azeem et al., 2014). Phosphorus buildup as phytate in soils can reach up to 51 million metric tons annually, accounting for 65% of phosphorus fertilizer. Phytase/phosphatase enzymes serve as essential mediators of organic phosphorus mineralization to use the soil’s organic phosphorus pool (Gaind and Nain, 2015). Protein and microbial-mediated degradation, partial or total incapacitation by adsorption onto soil particles and interaction with metal ions, microbial metabolites, and polyvalent anions are all important factors influencing phytase activity (Tang et al., 2006; Nannipieri et al., 2011).

Manures of monogastric animal and plant tissues and microbial conversion of soil inorganic phosphorus can all produce phytate (Liu et al., 2022). Phytase hydrolyzed the soluble forms of calcium and magnesium inositol phosphates; moreover, extracellular phytase activity has been identified in various plant species under phosphate stress situations. According to Wang et al. (2022), Mg-Al layered double hydroxides (LDHs) were found to be environmentally friendly materials to reduce phytate loss and promote the sustainable consumption of phytate when applied to the soil. Phytate or low phosphates are utilized by plants, which are present in soil and the utilization is enhanced when the soil contains phytase-producing microorganisms (Rao et al., 2009). Phytases provide a diverse role in various disciplines, but their major effects were studied in soil sustainability and feed additives.

## Role of microbial phytases in soil sustainability

**Figure 5:**
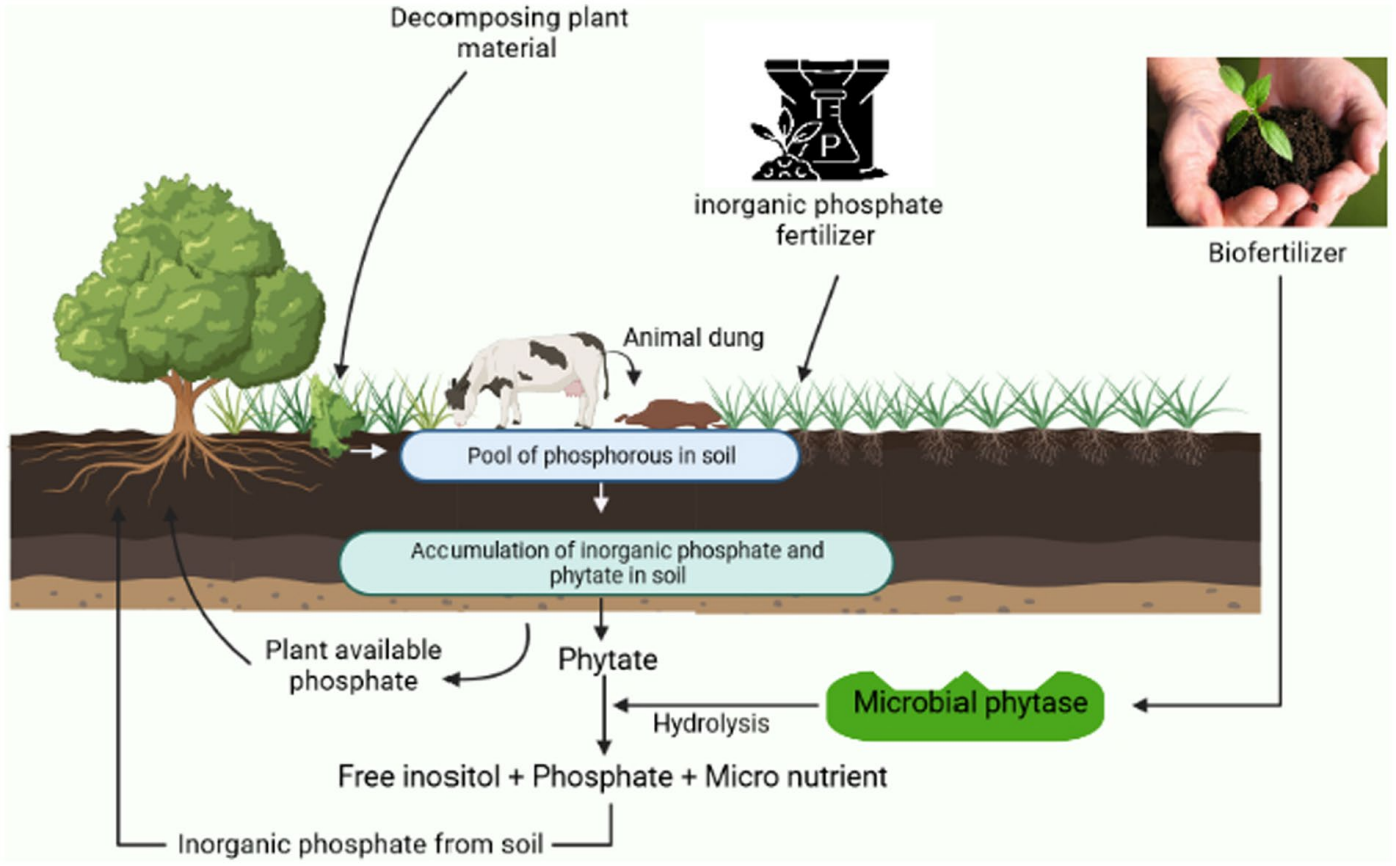
An overview to demonstrate the cyclic working process of a biofertilizer and the role of microorganisms in phytate solubilization.

Soils are formed through the dissolution of rocks and the minerals contained within them, and except for carbon, hydrogen, oxygen, and some nitrogen, soil serves as the environment for the growth of roots, and plants rely on soil for all other nutrients and water. Earlier, only the soil’s physical and chemical characteristics were thought to be significant. Yet, it is now well acknowledged that soil biodiversity plays a crucial role in preserving fertility and that soil biological activities are influenced by its physical and chemical properties. By the biotic operations of the microorganisms, the phytate can be found in soils in a variety of ways, including adsorbed to clays, as insoluble iron, calcium, and aluminum salt precipitates in acidic soils can be broken to the available form of phosphorus. Phytic acid also binds to the positively charged moiety of amino acids limiting their absorption; therefore, the proteins of leguminous plants consisting of more basic proteins are more readily bound to that of wheat proteins (Dersjant-Li et al., 2019). Phytases, from diverse sources in soil, are abundantly generated by numerous fungi, yeast, plants, animals, and bacteria, are necessary to hydrolyze phytic acid in the soil and are in charge of releasing phosphorus in rhizospheric regions of soil. The consumption of phosphorus from phytate is significantly influenced by the exogenous phytase activity of the roots of transgenic plants (Singh and Satyanarayana, 2011). There exist several phenomena of phytate utilization as discussed in the previous section “Mechanism of phytate solubilization.” The degradation of phytates produces many by-products and final products that enhance soil health (Figure 5). Phytase activity of a microbial source can be induced using optimized substrate and inoculum levels, pH, temperature, nitrogen and carbon additives, and resistance and sensitivity to the various metal inhibitors implied (Sadaf et al., 2022).

## Application of phytase-producing biofertilizers

High agricultural efficiency is based on lower synthetic chemical fertilizer doses and crop production costs. Phosphorus is supplied to crops in agricultural farming systems using produced phosphatic compounds. Because of their inherent value and possible agronomic capacity for plant development during prolonged phosphorus deprivation, bioinoculants may be a solution; phytases of microbial origin have emerged as an attractive target for commercial application (Kaur, 2020). Although several phosphate-solubilizing biofertilizer agents have been identified, they are primarily aimed at mobilizing rock phosphates in soil (Mohite et al., 2022).

Microbial phytases are thought to be a precise method for improving plant growth and productivity on a global level. Biofertilizers are regarded as very successful alternatives to synthetic fertilizers due to their simplicity, non-toxicity, environmental friendliness, and cost-effectiveness (Mazid and Khan, 2015). Biofertilizers have been demonstrated to increase the growth and development of plants by increasing the availability of macro and micronutrients to the plant system. *Azotobacter*, *Azosporillum*, *Phosphobacteria*, and *Rhizobium*-based biofertilizers are well-known and successful (Jorquera et al., 2008; Singh et al., 2014; Balogun et al., 2021; Chakraborty and Akhtar, 2021; Taj and Mohan, 2022). *Pseudomonas*, *Aspergillus* (*A. niger*, *A. flavus*, and *A. fumigatus*) (Balogun et al., 2021), *Burkholderia*, *Advenella* species, *Cellulosi microbium* sp. PB-09 (Singh et al., 2014), *Enterobacter,* and *Pantoea* isolates may release inorganic phosphate from phytate, thereby supporting agricultural sustenance. Microbial phytases are an appealing target for biofertilizers because they play an essential part in the soil phosphorus nutrient cycle. Many phosphate-solubilizing biofertilizers have been discovered, although their main function is to mobilize rock phosphates in soil. Biofertilizers are required to solubilize immobilized phosphate forms, like phytate, into mobilized form (Mohite et al., 2022). Phytases are essential for maintaining the balance of phosphorus in the environment, especially in its organic form, phytic acid, which reduces the need for chemical fertilizers to make up for phosphorus shortfall. Natural soil bio-resources, such as soil microorganisms, can be a viable alternative to traditional inorganic fertilizers. Some of the potential phytase-producing microbial strains with plant growth-promoting attributes are summarized in Table 1.

**Table 1:** Microorganisms producing phytase and having PGPR effects.

Microbial source	Microorganisms	Functions	Reference
Bacteria	*Klebsiella variicola*	Antioxidants Metabolites	Kusale et al. (2021)
	*Cedecea davisae*	Production of IAA, Ammonia, and phytase and solubilization of inorganic zinc and phosphate	Mazumdar et al. (2020)
	*Rahnella aquatilis* JZ-GX1	Promotes Seed Germination and Growth	Li et al. (2021)
	*Bacillus amyloliquefaciens* SQR9	IAA production	Shao et al. (2015)
	*Proteus mirabilis* BUFF14	Enhanced seed germination	Dhiman et al. (2019)
	*Bacillus clausii*	Produce lytic enzymes, Siderophores and solubilize inorganic phosphate.	Oulebsir-Mohandkaci et al. (2021)
	*Streptomyces* sp. (NCIM 5533)	Production of IAA, Ammonia, and phytase and solubilization of inorganic phosphate	Puppala et al. (2019)
	*Bacillus subtilis*	Enhancement of the growth performance of *Arabidopsis* and tobacco	Belgaroui et al. (2016)
	*Bacillus aryabhattai* RS1	IAA, ammonia, HCN, and siderophore production	Pal Roy et al. (2017)
	*Burkholderia* sp. AU4i	Promotes root and shoot elongation in pea	Usha et al. (2015)
	*Pseudomonas* sp. strain PSB-2	Solubilizing tricalcium phosphate	He and Wan (2022)
	*Arthrobacter* sp. strain PSB-5	Solubilizing tricalcium phosphate	He and Wan (2022)
Fungi	*Discosia* sp. FIHB 571	Production of siderophores, and biosynthesis of IAA- like auxins	Rahi et al. (2009)
	*Penicillium* spp. GP15-1	Stimulates growth and disease resistance	Hossain et al. (2014); Banerjee and Dutta (2019)
	*Aspergillus awamori*	Growth and seed production	Kour et al. (2019)
	*Aspergillus niger*	Solubilize the rock phosphate and make it available to plants	Din et al. (2019)
	*Trichoderma* sp.	Shoot and root growth	Kour et al. (2019); Cangussu et al. (2018)
	*Rhizopus arrhizus* KB-2	Stimulate plant growth	Evstatieva et al. (2020)
Yeast	*Candida tropicalis* HY	Stimulate rice seedling growth by production of IAA and ACC deaminase activity, deproteinization potential	Puppala (2018)
	*Saccharomyces cerevisiae* NCIM 3662	Hydrolyze phytate, probiotic and fortification properties	Puppala (2018)

## Phytase-producing transgenic plants

**Figure 6:**
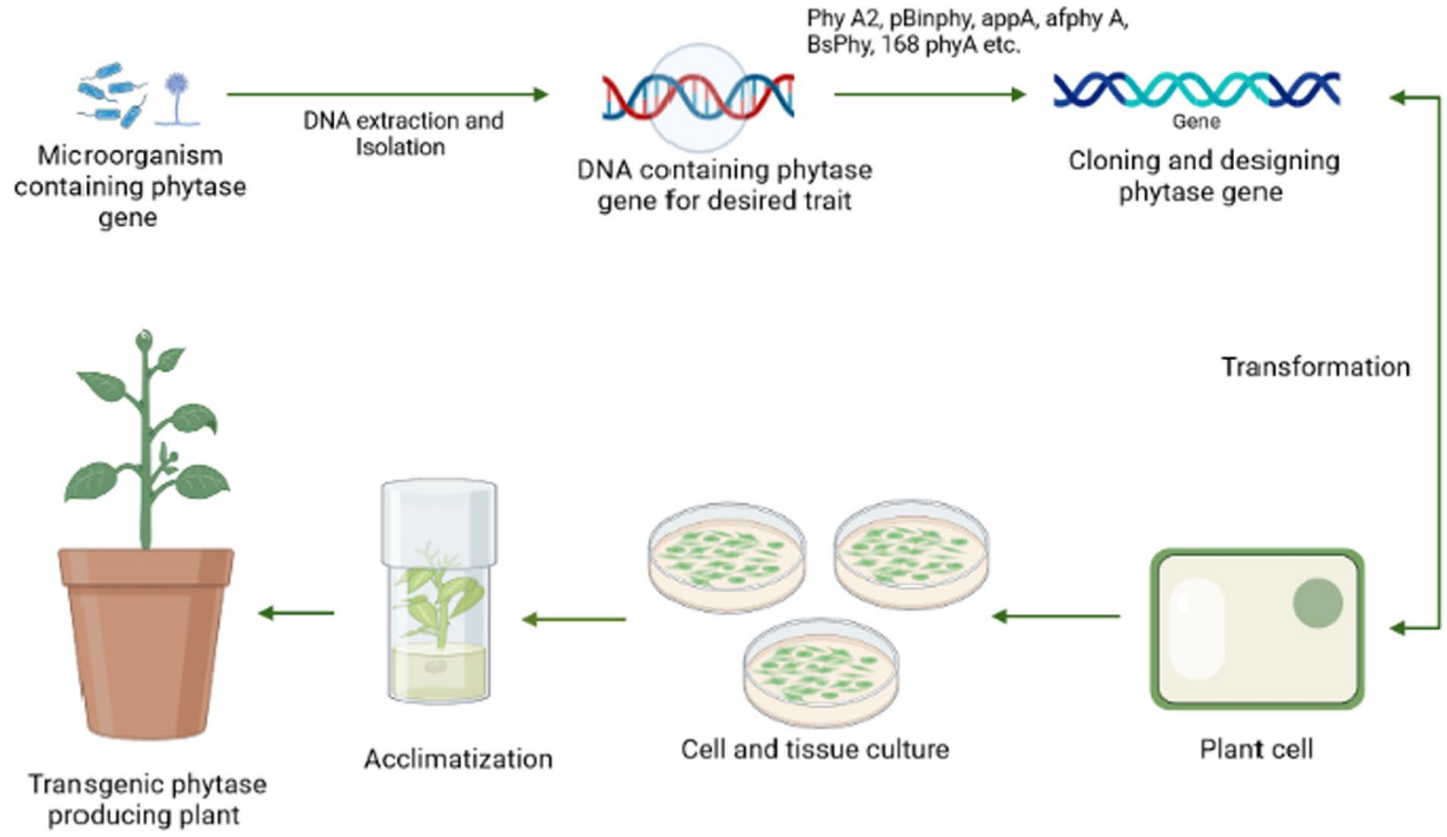
A pictorial representation of the steps involved in the generation of a transgenic plant beginning with the screening of an efficient microorganism possessing phytase gene followed by DNA extraction, cloning of phy gene, and transformation to the plant cell, which is further tested *in vivo* by plant tissue culture and then finally implemented to the field.

By maximizing the use of soil-based phosphate pools, including residual phosphorus, biotechnologies must be applied to agriculture to increase the efficiency of phosphate usage in crop production. For instance, intercropping cereals and legumes has been recommended to boost crop yields (Yang and Yang, 2021). Moreover, rhizosphere modification for phytate absorption may be ineffective because of low environmental fitness, inadequate metabolite production, or inoculum variability (Shenoy and Kalagudi, 2005). With the aid of modern technology, phytase can now be genetically inserted into crops (transgenic plants), used as biofertilizers, or added to the soil as pure enzymes. This also applies to all applications of phytate as a source of high phosphorus for animals, especially farm animals. The expression of regulating microbial phytase gene resulting in nutritious transgenic plant varieties might bring a significant outcome to resolve soil phytate consumption issues, as well as a lower dependency on external rock phosphate supply or biofertilizer treatment (Singh and Satyanarayana, 2011). The general mechanism for the transgenic development procedure has been stated in Figure 6.

In the last decade, the genes involved in the synthesis of microbial phytases having a high affinity toward phytate have been utilized to produce transgenic plants. Phytase genes from bacteria, fungi, and yeasts such as *B. subtilis, Selenomonas ruminantium, E. coli, Aspergillus ficuum*, *A. niger*, and *Thermomyces lanuginosus* have been used to develop transgenic plants. The most studied *A. niger* enzyme has been successfully expressed in *Arabidopsis*, tobacco, wheat, maize, soybeans, alfalfa, and canola (Valeeva et al., 2018). Several studies back up the advantages brought forth by transgenic types, such as the high expression of a PHY US417-related gene in *Arabidopsis* that led to increased growth and inorganic phosphorus content without inducing inorganic phosphorus starvation-triggered (PSI) genes. Enhanced biomass and Pi were seen in plants co-cultured with ePHY overexpression when the PSI gene was suppressed (Belgaroui et al., 2016).

*Bacillus* phytase production in tobacco cell cytoplasm alters the balance of inositol phosphate biosynthesis, providing more accessible phosphate (Singh and Satyanarayana, 2012). *Arabidopsis thaliana*, a transgenic plant grown solely on phytate, demonstrated better growth, which was related to root overexpression of the *A. niger* histidine acidic phosphatase gene phyA. Using *Bacillus* phytases, researchers have acquired further positive results in a transgenic plant (Balaban et al., 2017).

Transgenic soybean plants grew faster and absorbed more P (Singh et al., 2020). Transgenic soy roots produced *A. ficuum* histidine acid phytase (afphyA), which had 3.5–6 times the catalytic activity as compared to wild-type control plants for inorganic phosphate (Li et al., 2009). Sapara et al. (2022) studied the effectiveness of phytase gene promoters in transgenic varieties of tomato and cucumber, which were known to enhance minerals and micronutrient concentration, thus supporting nutrigenomics. Some of the transgenic varieties obtained are tabulated in Table 2.

**Table 2:** Transgenic plants developed in response to positive outcomes.

Source of microorganism	Gene	Plant	Reference
*A. niger*	*PhyA2*	Maize	Hu et al. (2022); Geetha et al. (2019); Tan et al. (2017)
*Aspergillus ficuum*	*PhyA2*	Maize	Jiao et al. (2021)
LBA4404 *Agrobacterium*	*PBINphy*	*Brassica napus*	Xu et al. (2020)
*Escherichia coli*	*appA (mappA)*	Soybean	Zhao et al. (2019)
*Aspergillus japonicas*	*PhyA*	Wheat	Akhtar et al. (2020); Cangussu et al. (2018)
*A. ficuum*	*AfphyA*	Soybean	Li et al. (2009); Balaban et al. (2017)
*B. subtilis phytase*	*BsPhy*	Cucumber	Sapara et al. (2022)
*E. coli*	*AppA*	Potato	Chen et al. (2020)
*A. niger*	*PhyA*	*A. thaliana*	Valeeva et al. (2018); Balaban et al. (2017)
*B. subtilis*	*168phyA*	*A. thaliana*	Valeeva et al. (2018); Lung et al. (2005)
*B. subtilis*	*168phyA*	Tobacco	Lung et al. (2005)
*Aspergillus niger*	*PhyA*	*Trifolium subterraneum*	Giles et al. (2017)

Despite these promising laboratory results, the situation in real-world soils would be less favorable. There is minimal evidence that such transgenic plants boost phosphate absorption and plant development in conventional soils; this has been supported by Blackburn (2012) in his experiments using *Trifolium subterraneum*, which resulted from better P absorption and more remarkable plant development found in agar was hampered when the same plants were exposed to the actual soil. One possible explanation is that phytases that have been released lose their action in soil due to adsorption. This rapid immobilization of the enzyme may limit the phytase’s capacity to interact with phytic acid in the soil, negating the previously predicted benefits of such a transgenic method to improve plant metabolism (George et al., 2004).

Bacterial phytases are frequently less expensive to produce and more accessible to express in plants than their eukaryotic counterparts. When using a bacterial phytase to make transgenic plants, it is critical to use an effective expression system inside the plants. Transgenic phytases are frequently introduced into the rhizosphere to aid in the decomposition of soil phytate and the improvement of soil phosphorus bioavailability, further increasing soil fertility and nutrient absorption. Plants engineered to express microbial phytase genes can release extracellular enzymes into the rhizosphere, enhancing phosphorus accumulation in plants. Phytases might reduce the risk of malnutrition while also lowering the phosphorus level of animal excrement. Phytases have enormous commercial and environmental potential. According to the existing literature, either the variation in phytase activity across plants has minimal impact on the phosphate nutrition of soil-grown plants or the baseline levels of phytase activity among plants have equal hydrolytic capacities. However, it is more likely that a significantly more considerable proportion of phytase obtained from microorganisms will disguise the variations in plant-exuded phytase (Liu et al., 2022). A remarkable study by Song et al. (2022) evaluated the effectiveness of the CRISPR/Cas9 mechanism to induce mutation within the phytate utilizing gene expression. They successfully reported the reduced phytic acid content with the deletion of specific gene segments.

## Conclusion and future prospective

Microbial phytases have been a need for the current scenario, as such naturally produced enzymes have various advantages, i.e., they are non-toxic, readily available, environmentally safe, and low production cost. Microbial phytases are among the most researched concerning their significance, action mechanism, and mass production. It has been hypothesized that certain phytases have positive health benefits, including heart disease, kidney disorders, and in some types of cancer. The food sector may potentially be interested in employing phytases to generate functional meals as well as to increase mineral bioavailability by lowering the phytate level in a particular product.

Consumers of agricultural goods are worried about their quality, nutritional value, and health. Phosphate solubilizing microorganisms (PSM) as inoculants is a measure for increased plant production. Although, their efficacy in replenishing the required plant nutrient is dependent on their establishment in the soil after competing for nutrients with natural flora. They are sensitive to plant pathogens due to the production of hydrogen cyanate, antibiotics, and antifungal metabolites. Their use is both environmentally and economically sound. Thus, using PSM as biofertilizers is a viable approach for increasing food production while posing no health risks and conserving the environment, and emerging the development of sustainable soil management. Genetic and protein engineering can be used to modify and improve the characteristics of enzymes. Transgenic plants and animals producing phytase and low-phytate crops are gaining popularity nowadays. Further advances in the creation of application-oriented phytases will usher in a new age in bioprocessing, broadening the scope of its efficiency and applicability. Furthermore, it emphasizes the use of advanced molecular techniques and genetic engineering to produce phytase genes of microbial origin for phytase synthesis. This strategy merits greater consideration since it can provide fresh research opportunities in future.

## Author contributions

BN: conceptualization, analyzed the data, written the original draft, and reviewed the manuscript. SR: analyzed the data and wrote the original draft. VijK: conceptualization, analyzed the data, wrote the original draft, and reviewed the manuscript. PEJS: analyzed the data and wrote the original draft. PS: analyzed the data and wrote the original draft. MC: analyzed the data and wrote the original draft. VivK, AV, and SM: writing—review and editing revision. All authors contributed to the article and approved the submitted version.

## Conflict of interest

The authors declare that the research was conducted in the absence of any commercial or financial relationships that could be construed as a potential conflict of interest.

## Publisher’s note

All claims expressed in this article are solely those of the authors and do not necessarily represent those of their affiliated organizations, or those of the publisher, the editors and the reviewers. Any product that may be evaluated in this article, or claim that may be made by its manufacturer, is not guaranteed or endorsed by the publisher.
